# Supplementary material for: Dietary protein shapes the profile and repertoire of intestinal CD4+ T cells
Source: J Exp Med. 2023 May 16;220(8):e20221816. doi: 10.1084/jem.20221816 (PMC10192604; doi:10.1084/jem.20221816)
Supplement: Table S1 — shows composition of protein antigen-free solid diet (AA). [file JEM_20221816_TableS1.docx]

**Table S1.** Composition of protein antigen-free solid diet (AA).

**Ingredients (%)**

Corn starch (35.71), Sucrose (25.90), Amino Acid Premix (17.57), Mineral Premix (10.00), Corn oil (5.00), Powdered cellulose (5.00), Sodium bicarbonate (1.00), Vitamin premix (0.20), Choline chloride (0.10), Ethoxyquin (0.02), DL-Alpha Tocopheryl Acetate (0.004)

| **Category** | **Component** | **% in Diet** |
| --- | --- | --- |
| **Protein** |  | **15.00** |
|  | Arginine | 0.58 |
|  | Histidine | 0.42 |
|  | Isoleucine | 0.79 |
|  | Leucine | 1.43 |
|  | Lysine | 1.20 |
|  | Methionine | 0.42 |
|  | Cystine | 0.06 |
|  | Phenylalanine | 0.79 |
|  | Tryosine | 0.83 |
|  | Threonine | 0.64 |
|  | Tryptophan | 0.18 |
|  | Valine | 0.94 |
|  | Alanine | 0.45 |
|  | Aspartic Acid | 1.06 |
|  | Glutamic Acid | 3.37 |
|  | Glycine | 0.32 |
|  | Proline | 1.95 |
|  | Serine | 0.91 |
|  | Taurine | 0.00 |
| **Fat** |  | **5.10** |
|  | Cholesterol | 0 |
|  | Linoleic Acid | 2.86 |
|  | Linolenic Acid | 0.05 |
|  | Arachidonic Acid | 0.00 |
|  | Omega-3 Fatty Acids | 0.05 |
|  | Total Saturated Fatty Acids | 0.64 |
|  | Total Monounsaturated Fatty Acids | 1.21 |
|  | Polyunsaturated Fatty Acids | 2.90 |
| **Fiber** | Cellulose | **5.00** |
| **Carbohydrates** |  | **66.20** |
| **Minerals** |  |  |
|  | Calcium | 1.21 |
|  | Phosphorous | 0.72 |
|  | Potassium | 0.41 |
|  | Magnesium | 0.01 |
|  | Sodium | 0.64 |
|  | Chloride | 1.1 |
|  | Fluorine | 0.0 ppm |
|  | Iron | 87 ppm |
|  | Zinc | 52 ppm |
|  | Manganese | 211 ppm |
|  | Copper | 5.0 ppm |
|  | Cobalt | 0.3 ppm |
|  | Iodine | 30.58 ppm |
|  | Chromium | 0.0 ppm |
|  | Molybdenum | 35.69 ppm |
|  | Selenium | 0.46 ppm |
| **Vitamins** |  |  |
|  | Vitamin A | 5.2 IU/g |
|  | Vitamin D-3 | 0.9 IU/g |
|  | Vitamin E | 20.0 IU/kg |
|  | Vitamin K | 2.00 ppm |
|  | Thiamin | 18.4 ppm |
|  | Riboflavin | 10.0 ppm |
|  | Niacin | 50 ppm |
|  | Pantothenic Acid | 28 ppm |
|  | Folic Acid | 4.0 ppm |
|  | Pyridoxine | 4.9 ppm |
|  | Biotin | 0.6 ppm |
|  | Vitamin B12 | 38 mcg/kg |
|  | Choline Chloride | 700 ppm |
|  | Ascorbic Acid | 250.0 ppm |
| **Energy** |  | 3.7 kcal/g |
